# ***ω***-3 PUFAs in the Prevention and Cure of Inflammatory, Degenerative, and Neoplastic Diseases 2014

**DOI:** 10.1155/2015/695875

**Published:** 2015-08-05

**Authors:** Karsten H. Weylandt, Yong Q. Chen, Kyu Lim, Hui-Min Su, Achille Cittadini, Gabriella Calviello

**Affiliations:** ^1^Division of Medicine, Department of Hepatology, Gastroenterology and Metabolism, Rudolf Virchow Hospital, Charité University Medicine, 13353 Berlin, Germany; ^2^Lipid Clinic, Experimental and Clinical Research Center (ECRC), Max Delbrück Center for Molecular Medicine and Charité University Medicine, 13353 Berlin, Germany; ^3^The Synergistic Innovation Center for Food Safety and Nutrition, State Key Laboratory of Food Science and Technology and School of Food Science and Technology, Jiangnan University, Wuxi, Jiangsu 214122, China; ^4^Department of Cancer Biology, Wake Forest School of Medicine, Winston-Salem, NC 27157, USA; ^5^Department of Biochemistry, School of Medicine, Cancer Research Institute, Infection Signaling Network Research Center, Chungnam National University, Daejeon 301-747, Republic of Korea; ^6^Department of Physiology, National Taiwan University College of Medicine, Taipei 100, Taiwan; ^7^Institute of General Pathology, Catholic University School of Medicine, 00168 Rome, Italy; ^8^Research Center for Biotechnology Applied to Cosmetology, Catholic University School of Medicine, 00168 Rome, Italy

While still a controversial topic, the omega-3 fatty acids as subject of research have come of age, particularly in recent years. They have made their way from being simple nutrition components, through possibly some exaggerations nominating them as universal tool to improve human health, to being the object of research in a wide variety of preclinical basic-research contexts as well as in smaller and larger clinical studies with mixed results regarding their potential health effects.

This is reflected in the wide variety of organ effects described in this special issue. The papers presented here range from the assessment of biological effects of omega-3 PUFA in vascular disease (M. Zanetti et al.; C. S. Bürgin-Maunder et al.) to their potential role in the prevention of Alzheimer's disease (T. Köhnke et al.), as dietary approach in the regulation of Helicobacter pylori-associated gastric diseases (J.-M. Park et al.), as combination treatment with antiestrogens for breast cancer prevention (A. Manni et al.), and to modulate apoptosis and proliferation in the placenta (E. Wietrak et al.). A particular focus of papers in this special issue is the lung, both regarding the acute respiratory distress syndrome (P. Cotogni et al.; M. Garcia de Acilu et al.) and regarding potential antitumor effects mediated by DHA effects on the mTOR/AMPK/PI3K-Akt pathway (N. Kim et al.).

This brings us to some of the most important issues in omega-3 PUFA research right now. This special issue presents several papers regarding mechanisms behind the biological effects of omega-3 PUFAs, in comparison to resveratrol in the context of inflammation, through their resolvin metabolites in the context of pain (J. Y. Lim et al.), as modulators of arachidonic acid lipid mediator cascade reactions (B. Lands), and through generation of biological active omega-3 PUFA derived electrophilic derivatives (C. Cipollina). It is probably the most important contribution of omega-3 PUFA research in recent years that a field of newly discovered and characterized metabolites from omega-3 PUFA has led our focus on metabolic products derived from omega-3 PUFA which are valid biologically active compounds by themselves.

Notwithstanding all these findings, there is also the role of the omega-3 PUFA as metabolic modulators and fuel for beta-oxidation. Both aspects, lipid metabolites modulation inflammation and metabolic effects of the fatty acids, are reflected in the context of obesity and hypercaloric diets and discussed in this special issue with regard to inflammation (Y. Wang and F. Huang) and mitochondrial function in the context of high fat diets (A. Ferramosca et al.). This focus on mitochondria then leads directly to the role of omega-3 PUFAs in energy metabolism, glycolysis, and the Warburg effect in cancer (L. Manzi et al.).

Finally, two papers address issues of omega-3 PUFA supply and administration, exploring approaches for heterologous reconstitution of these PUFAs in* Arabidopsis* (S. H. Kim et al.) and the pharmacokinetic profile of administering eicosapentaenoic acid (EPA) as free fatty acid in a stomach-protective capsule formulation (E. Scaioli et al.).

Given all these lines of research, the concluding review in this special issue then aims at the outline of a way forward for human studies with omega-3 PUFAs, in order to address and subsequently eliminate the uncertainties that are currently present in this exciting field of biomedical research (K. Weylandt et al.).

*Karsten H. Weylandt* *Yong Q. Chen* *Kyu Lim* *Hui-Min Su* *Achille Cittadini* *Gabriella Calviello*

